# Shaping high-performance wearable robots for human motor and sensory reconstruction and enhancement

**DOI:** 10.1038/s41467-024-46249-0

**Published:** 2024-02-26

**Authors:** Haisheng Xia, Yuchong Zhang, Nona Rajabi, Farzaneh Taleb, Qunting Yang, Danica Kragic, Zhijun Li

**Affiliations:** 1 School of Mechanical Engineering, Tongji University, Shanghai, 201804 China; 2 Translational Research Center, Shanghai YangZhi Rehabilitation Hospital (Shanghai Sunshine Rehabilitation Center), Tongji University, Shanghai, 201619 China; 3 Institute of Artificial Intelligence, Hefei Comprehensive National Science Center, Hefei, 230026 China; 4 Robotics, Perception and Learning Lab, EECS at KTH Royal Institute of Technology Stockholm, 114 17 Stockholm, Sweden; 5 Department of Automation, University of Science and Technology of China, Hefei, 230026 China

**Keywords:** Biomedical engineering, Electrical and electronic engineering

## Abstract

Most wearable robots such as exoskeletons and prostheses can operate with dexterity, while wearers do not perceive them as part of their bodies. In this perspective, we contend that integrating environmental, physiological, and physical information through multi-modal fusion, incorporating human-in-the-loop control, utilizing neuromuscular interface, employing flexible electronics, and acquiring and processing human-robot information with biomechatronic chips, should all be leveraged towards building the next generation of wearable robots. These technologies could improve the embodiment of wearable robots. With optimizations in mechanical structure and clinical training, the next generation of wearable robots should better facilitate human motor and sensory reconstruction and enhancement.

## Abstract (web summary)

Wearable devices and exoskeletons can restore and augment the user’s capability but are not necessarily perceived them as part of their bodies. In this perspective, the authors reflect on the importance of integrating environmental, physiological, and physical information to improve the embodiment of wearable robots.

## Introduction

Wearable robots are human-centered interdisciplinary systems incorporating diverse technological domains such as machinery, electronics, material science, computer science, integrated circuits, and control theory. These systems integrate robotic components with the human body, either in the form of exoskeletons worn atop the human body to enhance its capabilities; or in the form of powered prostheses that directly replace the lost limb functionality. They are designed to combat deficiencies resulting from neuro-muscular diseases or the loss of limbs. In addition to motor dysfunction, this deficiency encompasses sensory loss, impacting an individual’s autonomy and social interactions. The requirements and expectations for exoskeletons and prostheses are different. Exoskeletons are mainly involved in motor and sensory enhancement, whereas prostheses are mainly for their reconstruction.

Currently, the available technologies for integrating wearable robots with the human body face numerous limitations, resulting in wearers not perceiving the wearable robot as a part of their own bodies. This suggests that enhancing the embodiment of wearable robots is necessary. Prosthetic embodiment quantifies a user’s combined feeling of ownership and agency^1^. Serino et al. investigated cortical maps for movement control and touch sensing in individuals with upper limb amputation who underwent targeted muscle reinnervation, aiming for prostheses embodiment^2^. Rognini et al. combined tactile and visual stimulation in upper limb prosthesis users to increase embodiment and decrease perceived phantom limb distortion^3^. Fritsch et al. found attenuation of touch sensation in prosthesis users, which could serve as an indicator of prosthesis embodiment^4^. Likewise, in the context of exoskeletons, Forte et al. argue that exoskeletons should be tailored and adjusted according to the spinal cord injury patient condition to increase embodiment^5^. Hybart and Ferris proposed that measures of embodiment such as electroencephalography, reaction time, and proprioceptive drift could serve as metrics for assessing exoskeleton success^6^. Currently, the embodiment of wearable robots represents a critical yet insufficiently explored area, particularly in terms of enhancing their integration with the user.

Many challenges in robotics hinder the embodiment of wearable robots. Poor intelligence and functionality are common factors in device rejection by users^7^. Human-robot interaction requires perceiving the environment information as well as human physical and physiological information. This is while existing wearable robots often rely on single or limited modal sensors, which might result in inadequate performance^8^. Although wearable robots are human-centered systems, not putting the human user in the control loop remains a major concern^9^. For instance, issues may arise if individuals wearing exoskeletons are unintentionally pulled or moved against their intentions. A lot of wearable robots mainly focus on the control of the robots while omitting sensory feedback to the human, lacking bi-directional interaction between the human and robot^10^. Additionally, artifacts caused by skin movement between the wearable robot and the human body can significantly affect the control accuracy, leading to a significant drop in usage period and eventual abandonment^11^. Indeed, skin stretching during movement makes it challenging to maintain a complete connection between the human and the robot. Moreover, the current interface for idiodynamics and sensation is limited by a deficiency in information acquisition, transfer, and processing, especially for novel deep-learning neural network-based control algorithms that require high computational power^12^.

Wearable robots have existed for many years, but it is only in recent years that significant breakthroughs have occurred. Multi-modal fusion^13^, human-in-the-loop control^14^, neuromuscular interface^15^, flexible electronics^16^, and biomechatronic chip^17^, represent examples of advancements that could profoundly and positively impact the interaction between wearable robots and humans (Table 1). However, due to their complex nature, only a few of these advancements have been tested with users, often involving a limited number of participants^18^. Currently, each of the mentioned breakthrough technologies is available, but fully integrating these technologies requires the collaboration of multidisciplinary experts. These breakthrough technologies are transforming traditional wearable robots, though we expect their application to enable significant improvement that ensures these solutions mature enough and become the new benchmark. The widespread adoption of these technologies in wearable robots is expected to drive their optimization and standardization, ensuring the effective embodiment of high-performance wearable robots for motor and sensory reconstruction and enhancement.

**Table 1:** Recent results in the five enabling technologies for wearable robots

Enabling technology	Article type	Focus	Application	Year	Ref
Multi-modal fusion	Research	Continuous knee angle prediction	Exoskeleton control	2023	Guo et al.^123^
	Review	Multi-modal fusion method	Human-robot interaction	2020	Xue et al.^8^
Human-in-the-loop control	Research	Impedance adaptation	Exoskeleton control	2023	Li et al.^124^
	Review	Optimization and control strategies	Wearable robot control	2022	Diaz et al.^20^
Neuromuscular interface	Review	Invasive neural interface	Motor and sensory restoration	2023	Shen et al.^27^
	Review	Sensory Feedback	Limb prostheses	2021	Raspopovic et al.^10^
Flexible electronics	Research	Electrophysiological monitoring	Sensing under sweat condition	2022	Xie et al.^80^
	Review	Tactile sensation	Robot sensing	2022	Liu et al.^125^
Biomechatronic chip	Perspective	Neuromorphic hardware	Somatosensory neuroprostheses	2024	Donati et al.^126^
	Viewpoint	Neuromorphic computing	Robot control	2022	Sandamirskaya et al.^17^

**Fig. 1:**
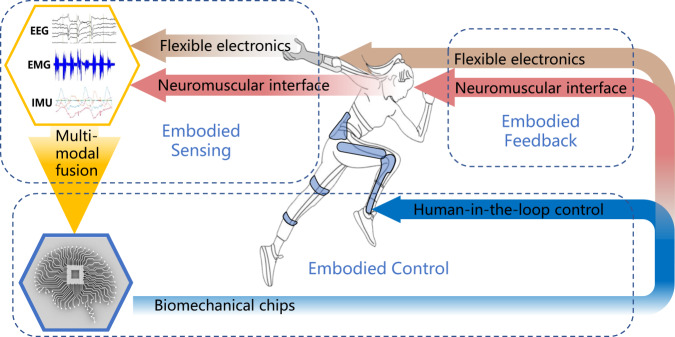
Breakthrough technologies for high-performance wearable robots. Various technologies of wearable robots for motor and sensory enhancement and reconstruction are depicted. Biomechatronic chips^127^ could serve as the central unit for information acquisition and processing, generating control commands. Neuromuscular interface^128^ and flexible electronics^129^ are enabling technologies for sensing human intention, which could be fused together with multi-modal fusion^13^. They also provide sensory feedback that transfers information from the robot to the human to improve the feeling of agency. Human-in-the-loop control^14^ integrates humans into the control loop of wearable robots, taking human reactions into account during the training process. (In the figure, arrows represent the information flow and dashed boxes represent technologies that form the key components of embodiment).

Researchers have extensively reviewed various papers on wearable robots, including exoskeletons for locomotor assistance^19^, human-in-the-loop optimization for wearable robots^20^, and sensory feedback for prostheses^10^. Unlike those papers summarizing the state-of-the-art in specific aspects of wearable robots, we propose a perspective that cutting-edge technologies of multi-modal fusion, human-in-the-loop control, neuromuscular interfaces, flexible electronics, and biomechatronic chips should be leveraged towards building high-performance wearable robots with embodiment. To narrow the scope to the embodiment aspect of wearable robots, we excluded the general aspects such as actuators, structures, electronics, and simulations. The embodiment of wearable robots mainly involves embodied sensing, embodied feedback, and embodied control, which led us to select the five enabling technologies: multi-modal fusion for embodied sensing; neuromuscular interface and flexible electronics for embodied sensing and embodied feedback; human-in-the-loop control and biomechatronic chips for embodied control. The logical relationship between the five technologies is around the embodiment of wearable robots (Fig. 1). Biomechatronic chips act as the central units for information acquisition, processing, and generating control commands. Neuromuscular interface and flexible electronics are enabling technologies for embodiment sensing of human intention, and embodiment feedback to improve the feeling of agency. Multi-modal fusion is utilized to improve the perception of human intention. And human-in-the-loop control integrates humans into the control loop with wearable robots, taking human reactions into account. We analyze how the development of these technologies would facilitate the development and verification of the next generation of wearable robots with a high embodiment. We propose a focused perspective on research progress and challenges in the five selected technologies for wearable robots, highlight their scientific foundations, and look into the potential future direction, to help researchers build a high-performance wearable robot with embodiment for motor and sensory reconstruction and enhancement.

## Motor enhancement

Integration of robotic components with the human body requires considerations in both technical and practical aspects, which is challenging when aiming at enhancing motor function. Failure to perceive changes in human states or the surrounding environment may result in delayed response or ineffective control. Not putting humans in the control loop restricts the interaction between robots and humans, and decreases the degree of participation. To surmount the challenges of integrating wearable robots with the human body, advanced technologies such as multi-modal fusion and human-in-the-loop control are being developed, augmenting the ability of robots to perceive the environment, and taking into account human intent.

### Multi-modal fusion

**Fig. 2:**
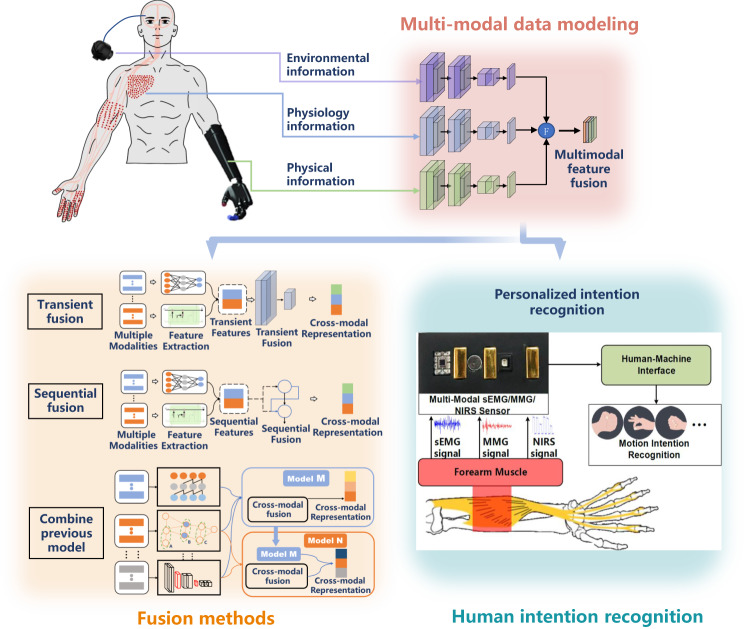
Multi-modal information fusion for wearable robots. Multi-modal signals were captured and extracted features, then fused with diverse fusion methods such as transient fusion and sequential fusion^8^. The fusion result was used to recognize human intentions and account for personal differences to achieve individuation^33^.

Given that wearable robots operate in unstructured environments and interact closely with humans, relying solely on information acquisition from a single sensor is significantly insufficient^21^. Consequently, incorporating multi-modal sensors enables wearable robots to accumulate and process information from diverse sources, leading to more precise and trustworthy motor enhancement. Just like human beings also utilize multiple modalities of sensing organs to perceive the world. Multi-modal information fusion helps wearable robots perceive the environment and recognize human intentions with diverse fusion methods, thus making the right decision upon motor enhancement operation to achieve compliant human-machine interaction (Fig. 2).

Single-modal information has demonstrated effective performance in capturing human intentions in wearable robot control. For example, high-density EMG provides detailed spatio-temporal information about muscle activity, enabling the decoding of intricate movements like hand gestures^22^. High throughput electroencephalography-based brain-computer interface (BCI) has achieved movement decoding for each finger^23^. Invasive BCI has also shown impressive performance in intention decoding. Longitudinal intrafascicular electrode has achieved prosthetic hand control^24^. High-density electrocorticographic (ECoG) can offer characteristic spatiotemporal neuronal activation patterns to decode hand gestures^25^, and has achieved exoskeleton control^26^. The invasive BCI can acquire high-quality neural signals, although the surgical risk and suboptimal long-term reliability hinder its widespread adoption^27^. Alternatively, people could choose many non-invasive approaches for intention decoding. Though one non-invasive signal alone is inferior to an invasive one, multi-modal information fusion could offer complementary details that potentially facilitate human intention recognition.

A pressure sensor combined with an inertial measurement unit (IMU) could provide information on ground contact and joint angles for wearable robot control^28^, though the control based on these signals is inherent behind the human physiological intention. Fusing electromyography (EMG) signals with an IMU has shown potential in prosthesis control, however, the EMG signals are susceptible to muscle fatigue which would influence the control accuracy^29^. Mechanomyography (MMG) signals accompanied by muscle movement have been proposed to be fused with EMG, which has enhanced human-exoskeleton interaction^30^. Ultrasonography and EMG have been combined, resulting in higher electrode-skin impedance compared to conventional EMG^31^, in which ultrasonography signals offer the morphological information of the muscle. Near-infrared spectroscopy (NIRS) has been used in conjunction with EMG and achieved a higher decoding performance in a prosthesis, compared to using each method individually^32^, in which NIRS adds the blood oxygen information during muscle movement. Sheng et al. integrated EMG, MMG, and NIRS for intention decoding, achieving the highest performance among all possible combinations^33^, in which MMG signals address the mechanical aspect of muscle movement. Chen et al. fused EMG, MMG, and ultrasonography to study the rectus femoris muscle during isometric contractions and found this combination could provide complete information on muscle contraction^34^.

The analysis of the muscle information may be limited by muscular functionality, as disabled elders often have very weak muscle signals. Electroencephalography (EEG) signals can be assumed to be consistently available. However, using EEG signals alone to control the wearable robot is not reliable, because these signals often have a low signal-to-noise ratio and are easily affected by artifacts. Combining EEG with EMG has shown great potential in improving the accuracy of detecting human intentions. For example, Kiguchi and Hayashi proposed a user’s motion estimation method for controlling wearable robots based on the user’s motion intention^35^, where EEG signals are employed as a compensatory measure in the absence of EMG signals. The combination of EEG and EMG was also employed for reconstructing the hand’s position in three dimensions^36^, in which EMG signals offer a wealth of movement-related information, and EEG signals provide supplementary data that enhances the reconstruction process. Environmental information is also important in human-machine interaction. For example, fusing vision gaze and EMG has been proven to enhance end-point control performance in upper-limb prostheses^37^. However, integrating high-throughput information like vision can result in extended processing times, placing a significant burden on the computing unit and potentially increasing latency.

Although we could choose from various modalities of information, employing a suitable information fusion method would warrant the enhancement of motor function in wearable robots. Transient fusion uses instant information and feeds it into the fusion module after preprocessing^8^, which is suitable for obtaining the current state of an object or human, for instance, instantaneous gesture recognition^38^. Sequential fusion introduces sequential data into a model with memory, such as long-short-term memory for multi-modal fusion to recognize human activity^39^. However, this kind of fusion involves sequential information stored in a memory, which might result in longer fusion times. To minimize the fusion time, the fusion method utilization of a previously trained model could play a role. Falco et al. achieved object recognition with tactile sensing fusion with a pre-trained visual model^40^. Although employing a pre-trained model could reduce the fusion time, it might sacrifice the output accuracy if the model is not fine-tuned on the new data. It should be noted that the inclusion of modalities increases the likelihood of failure. For example, the communication between modules for measuring different modalities becomes a major issue, along with the concerns associated with powering and calibrating them. Thus, despite the achievements made, current multi-modal fusion methods still need to improve time efficiency and accuracy simultaneously to satisfy the complex tasks of wearable robot control.

### Human-in-the-loop control

**Fig. 3:**
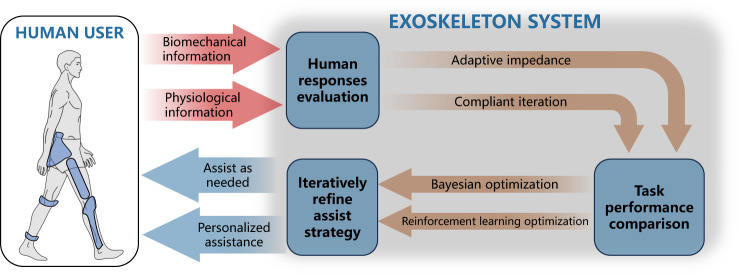
Human-in-the-loop control. In the process of using wearable robots, human biomechanical or physiological responses could be evaluated, and task performance could be compared over time. By using optimization methods, the wearable robot’s control strategy could be iteratively updated to optimization using effect.

As wearable robots are designed to be human-centric systems, incorporating the human element in the control loop is essential for effective operations (Fig. 3). However, existing wearable robots lack user interaction, leading to a mismatch between the user’s responses and the robot’s actions. This disconnection hinders the users’ ability to fully leverage the robot’s capabilities to enhance their motor functions. Human-in-the-loop control iteratively updates the controller parameters by considering the user’s response such as muscle activity, synergy, metabolic cost, gait symmetry, user preference or comfort, aiming to minimize or maximize that response^20^. It achieved a maximum reduction of energy cost by 37.9% for ankle exoskeleton^41^. The main drawback of this method is its tendency to require long iteration time thus inducing fatigue and increasing human dropout rate^20^.

Effective human-robot interaction is critical for optimal performance. A patient-cooperative control termed assist-as-needed provides torque assistance only if there is a large deviation from the intended movement^42^, which is an early-stage concept of human-in-the-loop control. Human biomechanical parameters such as joint angles, gait symmetry, and moving speed are some intuitive responses used in human-in-the-loop control^20^. However, this method aims to have the wearers achieve normative biomechanical parameters derived from previous clinical analysis^43^, which might not actually meet the needs of different individuals with different motor and sensory dysfunctions. To deal with this, Huang et al. developed a dynamic movement primitive to model motion trajectories of exoskeletons that updates iteratively to account for inter-subject preference^44^. Metabolic energy cost^45^ is a physiological parameter that is one of the most commonly used human responses in human-in-the-loop control, where wearable robots help humans to minimize movement effort. One drawback of this parameter is that it is usually calculated indirectly from oxygen intake and carbon dioxide output with empirical formulas which require several minutes of breath data, which is a lengthy time for updating control parameters. Regarding this, Gordon et al. achieved online metabolic cost estimation with a musculoskeletal model to save the sampling time^46^. Besides these parameters, muscle activity^47^, muscle synergy^48^, and user preference^49^ also have played a role in human-in-the-loop control.

Optimization is another key factor in the effectiveness of human-in-the-loop control. Advances in optimization strategies can offer optimal cooperation with humans by augmenting or reducing the above-mentioned variables. Evolutionary strategy is frequently used in human-in-the-loop control as it can manage many objective functions^41^. However, objective functions based on measurements of human performance typically require a mass of online and offline calculations, making it a lengthy evaluation period^50^. To improve the optimizing efficiency, Bayesian optimization has been utilized in human-in-the-loop control and achieved peak and offset timing of hip extension assistance identification eventually minimizing the energy expenditure^9^. This method has high time efficiency as it works by learning the shape of the objective functions to find parameters that can improve the result to the global maximum, while it becomes poor in efficiency when the number of objective functions and iterations increase. Reinforcement learning is a method learned by trial and error, where the good performance of humans is remembered and rewarded to facilitate human-robot interaction^51^. However, just because of its trial and error characteristic, there is a risk that the robot might apply harmful torque to the wearer^52^. Despite the achievements, most optimization algorithms are heavy on computation, which brings new challenges to the calculation power of the wearable solution.

## Sensory reconstruction

Sensory reconstruction builds a new pathway for delivering sensory information from the robot to the human. As motor and sensory functions are strongly integrated as a fundamental principle of human beings, the reconstruction of motor function should not develop alone from the integration of sensory reconstruction. An ideal wearable robot should thus reconstruct and enhance the motor and sensory functions of the dysfunctional body part. However, in practice, rebuilding sensory feedback is extremely challenging^10^.

### Neuromuscular Interface

**Fig. 4:**
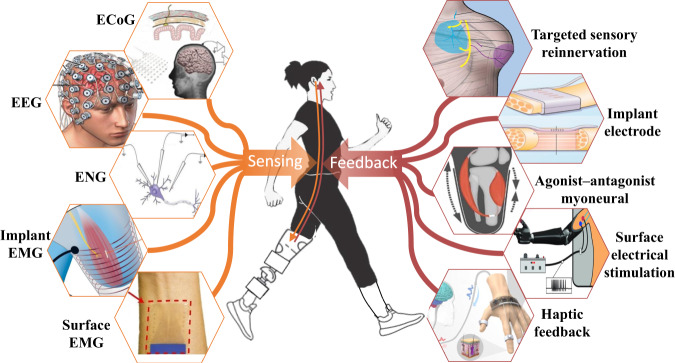
The bidirectional neuromuscular interface. The sensing interface captures signals on the efferent nerve pathway (orange arrow downwards), which could be used to sense human intention, for example, ECoG^130^, EEG^131^, electroneurogram (ENG)^132^, implant EMG^133^, and surface EMG^134^. The feedback interface stimulates the afferent nerve pathway (indicated by the upward red arrow), which could be used to convey information to humans, for example, in non-invasive ways such as haptic feedback^67^ and surface electrical stimulation^70^, in implantable ways like agonist-antagonist myoneural^76^, implant electrode^72^, and targeted sensory reinnervation^77^.

The neuromuscular interface functions bidirectionally, including sensing and feedback. To sense human movement intentions, sensing neuromuscular interface through the nerves or muscles can be probed directly. The sensory feedback neuromuscular interface transmits information about the external environment or human body state to the nervous system to build a feedback loop (Fig. 4). The sensory feedback interface is crucial for wearable robots, particularly during tasks requiring physical interaction with objects. For example, a prosthesis equipped with sensory feedback can restore lost perception to an amputee, while an exoskeleton with sensory feedback can convey information about the contact force with the environment to the wearer.

The sensing interface captures signals along the efferent neural pathway to establish a connection between humans and wearable robots. For example, BCI can use brain signals from the scalp, the cortical surface, or intracortical to restore movement control to paralyzed people^53^. ECoG signals were for used chronic neural recording and stimulation^26^. The invasive brain-computer interface has high control accuracy while most people could not accept brain surgery, and the non-invasive brain-computer interface typically has relatively lower accuracy^54^. Recent years have witnessed significant advancements in non-invasive techniques^55^, such that non-invasive BCIs have demonstrated improvements in motor imagery tasks^56^. EEG signals have been investigated as an alternative for measuring brain activity and conveying user intention to wearable robots^57^. The control process typically involves decoding EEG responses elicited by imagining the intended task such as gait^58^ or manipulating objects^59,60^. Surface EMG is the most commonly used neuromuscular interface for sensing human intent in wearable robot control^29^, while it is susceptible to electrode placement and skin conditions. High-density EMG provides detailed spatio-temporal information about muscle activity, enabling the decoding of intricate movements like hand gestures^22^. Implant EMG could solve the problem of surface EMG and improve robustness in wearable control^61^. This method is confined when in the case of amputation not enough remnant muscle tissue is left for implanting electrodes. ENG is a neural electrical signal^62^ that requires a nerve implant and could handle the above-mentioned problems for both invasive and non-invasive EMG interfaces, and has demonstrated promising results in motor intention decoding^63^. However, this method has problems of poor signal-to-noise ratio and stability^64^, and might just resemble the decoding result similar to surface EMG^65^. Besides, all implants in the brain, nerve, or muscle have the concern of durability^66^. Thus, despite the advances, current sensing neuromuscular interfaces still need to improve accuracy for non-invasive approaches and durability for invasive ones.

In the afferent nerve pathway, feedback plays a vital role in enabling wearable robots to effectively communicate with the user, facilitating intuitive interactions. Vibration feedback to generate tactile on the skin is a commonly used way to provide haptic sensation^67^. Though, in theory, vibration could be modulated to different frequencies and intensities to map different sensations^68^, it would enforce a cognitive burden for the user to master the relationship. Iberite et al. used wearable devices to restore natural thermal sensory feedback in individuals with amputation^69^. Electrical stimulation with current into the skin could generate electrotactile^70^. However, this method is usually rated by users as tingling^71^. External stimulations are inherently less intuitive compared to internal nerve stimulation, which could activate the same sensation pathway^72^. Long-term stimulation of the nerve might lead to decreased sensitivity^73^ and make it hard for information to be conveyed. However, a recent clinical trial has shown the feasibility of the six-month use of hand prosthesis with intraneural tactile feedback^74^. Valle et al. have demonstrated that intraneural sensory feedback improves sensation naturalness, tactile sensitivity, and prosthesis embodiment^75^. Agonist–antagonist myoneural interface could naturally convey proprioception for prosthesis user^76^, while it is confined to the position of amputation. To deal with that, targeted sensory reinnervation could achieve sensation by simulating other parts of the body^77^. Significant research progress has been shown in the self-contained hand prosthesis with sensory feedback over 3–7 years of use in 4 individuals with transhumeral amputation^78^. Similar to sensing, sensory feedback could also consider multi-modal integration to improve naturalness, such as thermal together with tactile feedback^69^. Current sensory feedback technology still needs to work towards creating a neural pathway that transfers a large amount of sensory information to the nervous system and ensuring an effortless experience for the user.

### Flexible electronics

**Fig. 5:**
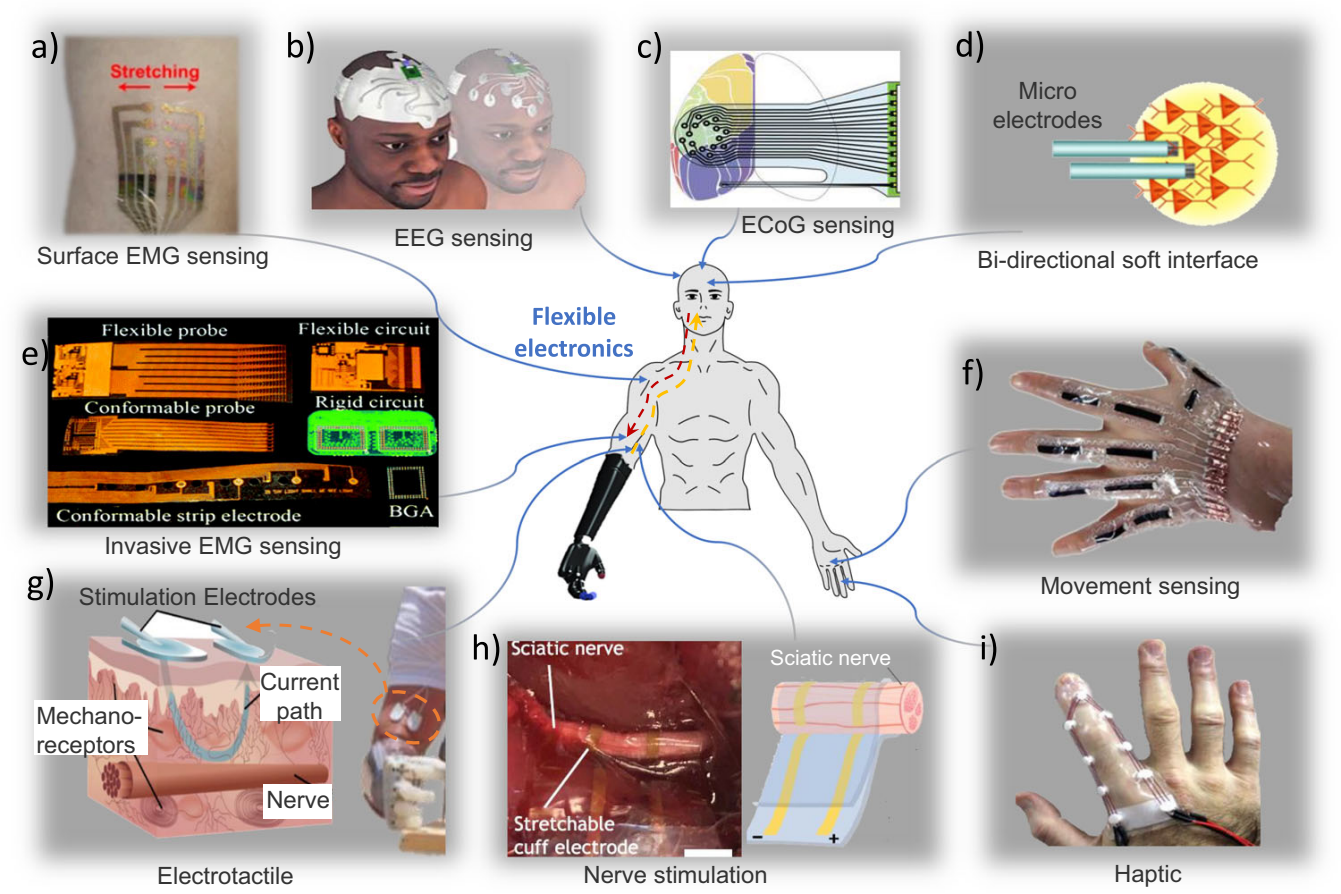
Flexible electronics for sensing and feedback. It could be conformal with human skin or internal organs to capture signals or deliver stimulations. **a** Flexible electronics for sensing surface EMG^80^. **b** Flexible electronics for sensing EEG^85^. **c** Flexible electronics for sensing ECog^86^. **d** Flexible electronics for sensing and feedback^93^. **e** Flexible electronics for sensing invasive EMG^83^. **f** Flexible electronics for sensing hand movement^87^. **g** Flexible electronics for providing electrotactile^89^. **h** Flexible electronics for nerve stimulation^90^. **i** Flexible electronics for providing haptic feedback^88^.

It is difficult for traditional rigid sensors to conform to the human skin or internal organs when measuring neuromuscular signals or delivering the sensory feedback mentioned earlier, which often results in motion artifacts and low measurement accuracy. As a person moves, the skin stretches, wrinkles and flexes, and internal organs beat, all with large deformation. This makes it difficult to maintain contact between the sensors and humans. The flexible electronics nature of soft and stretchable, minimizing the physical and mechanical mismatch between skin/neural tissue and the flexible electronics^79^, potentially enabling a high-quality interface (Fig. 5).

Flexible electronics have the potential to form the bidirectional neuromuscular interface discussed previously. For neuromuscular signal sensing, Xie et al. developed a nano-thick porous stretchable dry electrode for surface EMG sensing, which even works under sweating conditions^80^. Hydrogel has been commonly used for electrophysiology signals in wearable robot applications for its skin-like properties and good conductivity^81^. However, the hydrogel sensor is limited in a humid environment, which could not withstand a long time in the air while this characteristic makes it a suitable interface for implant electrodes^82^. Poly (3,4-ethylenedioxythiophene) (PEDOT) material has been used to form implant EMG electrodes^83^. Liquid metal has achieved implant ENG signal recording^84^. For brain signals, Carneiro et al. developed a headband with conductive stretchable ink for forehead EEG signal acquisition^85^. Invasive ECoG has also been shown to be recorded by flexible electronics^86^. Besides, flexible electronics could sense human mechanical movement with multiple channels^87^. Spatial resolution is important for sensing neuromuscular signal quality. Thus, flexible electronics for wearable robots need to form high-density arrays and overcome difficulties in wiring, especially during strain when the connection tends to break^80^.

Flexible electronics may also function as an interactive feedback mechanism, enhancing the communication between humans and wearable robots. Chossat et al. have developed a soft skin stretch device with twisted and coiled polymer for generating haptic feedback^88^. Akhtar et al. used a flexible interface to achieve electrotactile touch feedback in prosthesis users^89^. For in vivo sensory feedback, flexible electronics have also played a role. Lienemann et al. developed cuff electrodes with stretchable gold nanowires and achieved invasive peripheral nerve stimulation^90^. Minev et al. developed an electronic dura mater capable of delivering electronic stimulation and even drugs^91^. Similarly to sensing, feedback also requires high resolution. Zhu et al. achieved large area pressure feedback with flexible electronic^92^. To meet the bi-directional requirements for the neuromuscular interface, flexible electronics need to integrate sensing and feedback. Vitale et al. achieved neural stimulation and recording with soft carbon nanotube fiber microelectrodes^93^. Since many flexible neuromuscular interfaces work in vivo, they need to head off toward high biocompatibility and long durability to avoid rejection and replacement.

### Biomechatronic chip

**Fig. 6:**
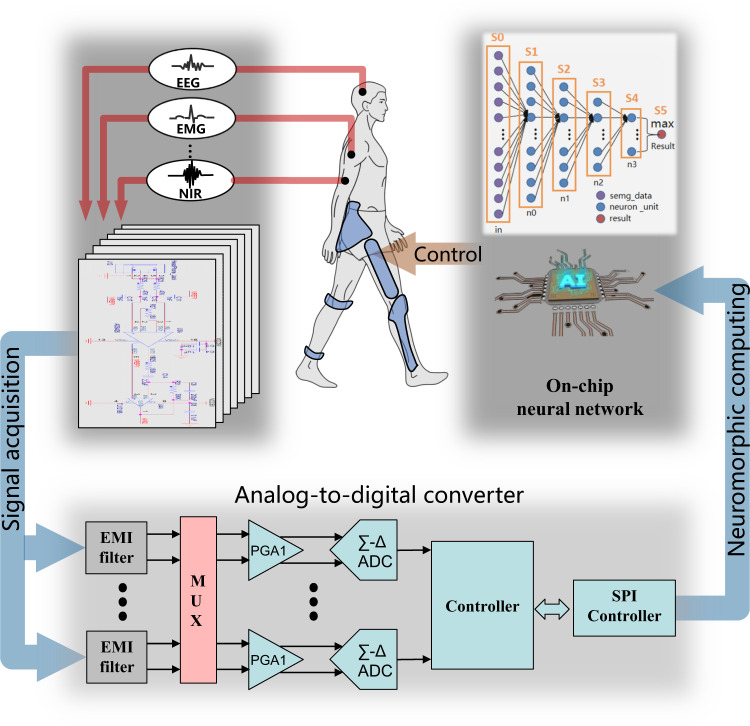
Biomechatronic chips for wearable robots. The signal acquisition part intakes multi-modal information, ADC prepares processable data, and neuromorphic computing handles on-chip neural network calculation^94^. The biomechatronic chip serves as the central control unit for the wearable robot.

For wearable robots, dealing with human physiological signals involves acquisition, transfer, and processing, while currently idiodynamic and sensation are limited by a deficiency in human-robot interface. As a weak signal, the human physiological electrical signal first needs to be amplified to meet the level of the following circuit and handle the problems such as electromagnetic interference, human artifact, and noise. Then, the analog-to-digital converter (ADC) directly determines whether the data used for signal processing is reliable. Meanwhile, the latest wearable robot control and optimization involves a large-scale deep learning network, which is extremely costly in conventional computing hardware (Fig. 6). Biomechatronic chip is specifically designed for the acquisition, transfer, and processing of biomechatronic information^94^, which is very attractive in acting as the central processing unit for a wearable robot.

In wearable robot applications, data acquisition faces challenges in a multitude of signal modalities, multi-channels, weak raw signal amplitude, and noises. To reduce noises in neuromuscular sensing, chopper modulation technology and current multiplexing technology have been proposed to modulate input noise when gathering neural signal^95^. For amplification of the weak neuromuscular signal, Ng and Xu applied single-ended CMOS-inverter-based preamplifiers for both the reference and neural signal inputs and achieved a low power^96^. For different modality signals, different signal amplification gains have been achieved with a programmable gain amplifier^97^. In consideration of the multi-channel data acquisition to increase signal resolution, Luo et al. designed a 16-channel neural-signal acquisition chip and achieved low noise^98^. Besides, to suppress the offset voltage of the neuromuscular signal, a direct current servo loop is introduced to reduce the offset^99^. As the front end, signal acquisition still needs to work towards optimizing the above-mentioned parameters simultaneously with low power consumption.

Analog-to-digital converter (ADC) is vital for biomechatronic chips in wearable robot applications as it determines the data quality used for subsequent signal processing. The primary consideration is the accuracy of conversion, Ahmed and Kakkar designed an auto-configurable successive approximation register ADC for neural implants and achieved a good performance^100^. However, the offset compensation that is important for ADC was not considered in this design. Wendler et al. developed a Delta-Sigma ADC with 120 mVpp offset compensation for neural signal recording^101^. Power consumption and number of channels are two main factors that limit the development of ADC for wearable robot applications. Gagnon-Turcotte et al. designed a CMOS biomechatronic chip for simultaneous multichannel optogenetics and neural signal recording and achieved a low power of 11.2uW^102^. However, the chip area would increase when the number of channels increases for the high density of the neuromuscular signal. Besides, the stimulation artifact should be rejected to achieve a bidirectional neuromuscular interface. Pazhouhandeh et al. proposed a neural ADC that achieves blind stimulation artifact rejection, where a bidirectional COMS neural interface could capture neural signals during neural stimulation^103^.

Current wearable robot control and optimization involve large-scale deep learning network algorithms, which require a high hash rate and even a dedicated hardware structure for neural network calculation. Mimicking the dynamics of spiking neurons and dynamic synapses, a neural morphology chip ‘Loihi’ was proposed that could conduct such computation efficiently with integrated circuits^104^. With the recent advance in material with memristive properties which holds the state induced by a transient spike, the neuromorphic chip could be efficient and compact in neural network computing^105^. Based on this, Kreiser et al. realized simultaneous localization and mapping with neuromorphic chips^106^, which holds the potential for wearable robots that assist visually impaired individuals. Stewart et al. achieved online gesture learning with a neuromorphic chip^107^, which could be suitable for prosthetic applications. Flexible neuromorphic electronics for neuroprosthetics are also achieved^108^. Despite these achievements, as algorithm development is faster than hardware, there is a limitation in reconfigurability in neuromorphic chips to construct different network architectures to adapt different wearable robot applications.

## The next generation of wearable robots

We foresee the performance of the next generation of wearable robots will broaden their applications by leveraging the breakthroughs - multi-modal fusion, human-in-the-loop control, neuromuscular interface, flexible electronics, and biomechatronic chip. With intuitive control and proprioception, next-generation wearable robots should better meet the user requirement for motor and sensory enhancement and reconstruction. The fusion of multi-modal information could guarantee the performance in perceiving the environment and human intention. Putting humans in the control loop in this human-centered system provides an intuitive human-robot interaction. The establishment of a bidirectional neuromuscular interface could reconstruct the neural pathway for sensation. Applying flexible electronics could handle artifacts caused by movement between the wearable robot and the human. Moreover, the adoption of biomechatronic chips with dedicated information acquisition, transfer, and neural network computation would empower fast signal processing for wearable robots.

Multi-modal fusion will eliminate the deficiency in perception of environment information and human intention information, especially for complex human intention. In order to use complementary characteristics of multi-modal information obtained from heterogeneous sensing, a novel multi-modal feature fusion strategy for human motion intention recognition shall be studied^109^. Besides, an accurate motion intention recognition model with the ability to adapt to different individuals with various motor and sensory dysfunction features is of great importance^5^. The fusion of available auxiliary modalities such as EMG signals, and visual images can enhance the performance of EEG-based BCI for wearable robots^110^. Moreover, the accuracy of human motion intention recognition and real-time performance determines the overall user acceptability of wearable robots. Advances in the fusion of multi-scale multi-modal information would warrant compliant human-machine interaction.

The next generation of human-in-the-loop control aims at developing control strategies that are simple and “fit like a glove” to each user, integrating information from multiple human-related responses to generate coherent control parameters configurations and improve human-robot interaction^111^. Despite the different needs of exoskeletons and prostheses, a natural and reliable control that minimizes the interaction forces between the robot and human to improve comfort is a desired characteristic for both types of wearable robots. Discomfort and problems with body fitting are common causes of device rejection^112^. It is also worth noting that the wrong set of control parameters may cause discomfort and pain^113^, thus personalization might be necessary to engage. Future control should consider the user preference, and coactive feedback and utilize cost functions together with other metrics to optimize interaction which increases users’ acceptance^114^. Advancements in closing the human-robot loop of wearable robot control strategies can benefit different populations with motor and sensory dysfunctions.

Wearable robots involving neuromuscular interfaces have demonstrated significant benefits in rehabilitation, while the widespread adoption of such wearable robots still faces significant challenges. For example, although BCI has shown promising results in detecting the user’s intentions while using a wearable robot their actual effectiveness is yet to be fully explored. While invasive brain imaging methods provide better accuracies, their acceptance is likely to be limited to a small group of patients as the concern of safety and durability of the invasive neuromuscular interface, which might cause unwanted surgery^115^. Conversely, non-invasive methods, such as EEG, contain less information and are more susceptible to noise and artifacts. Therefore, better recording hardware and detection algorithms are still required to prepare EEG-based BCI for real-world applications. Another challenge in using neuromuscular interfaces is the long adaptation time. Due to the signal’s variability across sessions and individuals, using the same BCI system without calibration or retraining is nearly impractical. Therefore, enhancing the transferability of learned representations is crucial for seamless integration of these systems with wearable robots. Besides, the neuromuscular interface typically builds on humans, while it is also important to build an interface between the robot and environment, for example, sensors on the prostheses for generating sensory feedback for humans^116^. In order to achieve a positive user experience in the use of wearable robots, the neuromuscular interface should include both reliable decoding of user intentions and real-time delivery of sensory feedback. In the future, the continuous development of bidirectional neuromuscular interfaces will bring leaps to wearable robots for motor and sensory reconstruction.

The flexible electronics offer the advantage of conformation to human skin or tissue. It also has good scalability and can be prepared into different shapes to meet the requirements of different wearable robots. The new generation of flexible electronics for wearable robots shall achieve stretch and healing itself, low cross-coupling, low-cost processing, and multi-sensor integration^117^. Self-power supply is another direction that could benefit the arrangement of interfaces for wearable robots^118^. One step forward, flexible electronics could not only serve as sensors but also as the interface for sensory feedback^119^. This integration would greatly benefit the above-mentioned bidirectional neuromuscular interface. Advances in flexible electronics will provide new strategies for the research and development of wearable robots.

The next generation of wearable robots should apply dedicated biomechatronic chips with high performance in data acquisition, ADC, and processing. As multi-modal signals have various levels of amplitudes, chips with programmable gain amplifiers which can be digitally controlled to obtain multiple amplification ratios to adapt to changes in the input signal would be highly beneficial^120,121^. Bit error is a problem in ADC, adding redundant bits to the binary code could improve system fault tolerance and reduce bit error probability, and bringing digital calibration technology into the system would improve the effective resolution^122^. Neuromorphic chips could run neural networks with various topologies and thus could support large-scale deep learning neural network algorithms, where the neuromorphic hardware empowers continual learning with training data and experience together. Those three key components constitute the next-generation biomechatronic chip that can enrich wearable robots with a powerful heart.

Realistically, the wider clinical application of multi-modal fusion, human-in-the-loop control, neuromuscular interface, flexible electronics, and biomechatronic chips should occur within the next decade for wearable robots. All of these technologies have been validated and shown to have benefits for people with motor and sensory dysfunction, such as hemiplegic patients and amputees. Exoskeletons and prostheses leveraging these cutting-edge technologies together will constitute a new generation of wearable robots. We expect them to significantly improve the users’ quality of life and pave the way for motor and sensory enhancement and reconstruction.
